# MiR‐503 pleiotropically regulates epithelial‐mesenchymal transition and targets PTK7 to control lung cancer metastasis

**DOI:** 10.1002/cam4.6116

**Published:** 2023-05-22

**Authors:** Tzu‐Hsiu Tsai, Chien‐Hung Gow, Yi‐Nan Liu, Meng‐Feng Tsai, Tzu‐Hua Chang, Shang‐Gin Wu, Min‐Shu Hsieh, Kang‐Yi Su, Jin‐Yuan Shih

**Affiliations:** ^1^ Department of Internal Medicine National Taiwan University Hospital Taipei Taiwan; ^2^ Department of Internal Medicine Far Eastern Memorial Hospital New Taipei City Taiwan; ^3^ Department of Healthcare Information and Management Ming‐Chuan University Taoyuan Taiwan; ^4^ Department of Biomedical Sciences Da‐Yeh University Changhua Taiwan; ^5^ Department of Pathology National Taiwan University Hospital Taipei Taiwan; ^6^ Department of Clinical Laboratory Sciences and Medical Biotechnology College of Medicine National Taiwan University Taipei Taiwan; ^7^ Graduate Institute of Clinical Medicine College of Medicine National Taiwan University Taipei Taiwan

**Keywords:** actin cytoskeleton, epithelial‐mesenchymal transition, focal adhesion kinase, miR‐503, protein tyrosine kinase 7

## Abstract

**Objective:**

In lung cancer patients, most deaths are caused by the distant dissemination of cancer cells. Epithelial–mesenchymal transition (EMT) and collective cell migration are distinct and important mechanisms involved in cancer invasion and metastasis. Additionally, microRNA dysregulation contributes significantly to cancer progression. In this study, we aimed to explore the function of miR‐503 in cancer metastasis.

**Methods:**

Molecular manipulations (silencing or overexpression) were performed to investigate the biological functions of miR‐503 including migration and invasion. Reorganization of cytoskeleton was assessed using immunofluorescence and the relationship between miR‐503 and downstream protein tyrosine kinase 7 (PTK7) was assessed using quantitative real‐time PCR, immunoblotting, and reporter assays. The tail vein metastatic animal experiments were performed.

**Results:**

Herein, we demonstrated that the downregulation of miR‐503 confers an invasive phenotype in lung cancer cells and provided in vivo evidence that miR‐503 significantly inhibits metastasis. We found that miR‐503 inversely regulates EMT, identified PTK7 as a novel miR‐503 target, and showed the functional effects of miR‐503 on cell migration and invasion were restored upon reconstitution of PTK7 expression. As PTK7 is a Wnt/planar cell polarity protein crucial for collective cell movement, these results implicated miR‐503 in both EMT and collective migration. However, the expression of PTK7 did not influence EMT induction, suggesting that miR‐503 regulates EMT through mechanisms other than PTK7 inhibition. Furthermore, we discovered that PTK7 mechanistically activates focal adhesion kinase (FAK) and paxillin, thereby controlling the reorganization of the cortical actin cytoskeleton.

**Conclusion:**

Collectively, miR‐503 is capable of governing EMT and PTK7/FAK signaling independently to control the invasion and dissemination of lung cancer cells, indicating that miR‐503 represents a pleiotropic regulator of cancer metastasis and hence a potential therapeutic target for lung cancer.

## Graphical abstract

MiR‐503 is a pleiotropic regulator of lung cancer metastasis, governing both epithelial‐mesenchymal transition (EMT) and EMT‐independent PTK7/FAK signaling.MiR‐503 may represent an attractive target for therapeutic interventions in lung cancer.

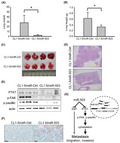

## 1 INTRODUCTION

Among all cancers, lung cancer is responsible for the greatest number of deaths.^1^ In 2020, lung cancer was diagnosed in approximately 2.2 million people worldwide and caused 1.8 million deaths. The five‐year survival rate for patients with advanced lung cancer is estimated to be only 10%–20% in most countries.^1^ In particular, lung cancer deaths are most commonly caused by metastasis of cancer cells resistant to antitumor treatments. Therefore, it is imperative to explore new paradigms of lung cancer metastasis in order to develop novel therapeutic strategies.

Whereas cancer dissemination relies on a series of interdependent steps, migration and invasion of tumor cells constitute an integral part of the metastatic cascade.^2^ It is known that epithelial cells either migrate cohesively or undergo epithelial‐to‐mesenchymal transition (EMT) to move individually as mesenchymal cells.^3^ EMT, an embryonic program to enable epithelial cells to gain a mesenchymal phenotype, is characterized by the expression of mesenchymal markers and the loss of cell‐junction proteins in the involved cells.^4^ Although there is debate regarding whether full EMT is required for the acquisition of drug resistance rather than for metastatic spread, EMT undoubtedly confers increased motility in individual cells and a vast body of literature correlates EMT with cancer metastasis.^5^, ^6^, ^7^ In contrast with the single‐cell migration associated with EMT, collective migration represents a different pattern of cell movement in that constituent cells migrate coordinately in multicellular clusters and maintain their cell–cell junctions and front‐rear polarity.^8^ Specifically, protein tyrosine kinase 7 (PTK7), a component in the Wnt/planar cell polarity (PCP) pathway, is pivotal for collective movements.^9^, ^10^, ^11^, ^12^ It is becoming evident that most epithelial cancers utilize collective migration, rather than EMT‐associated mesenchymal migration, as the primary mean for cell invasion and metastasis.^13^ However, EMT and collective migration are not necessarily mutually exclusive in cancer dissemination. For example, it has been shown that cancer cells undergoing partial or hybrid EMT move in a manner of collective migration.^14^, ^15^, ^16^ Furthermore, cancer cells in the leading front during collective migration were found to acquire a more mesenchymal phenotype through EMT.^17^, ^18^ These observations indicated that these two distinct processes may be interrelated in cancer invasion and metastasis.

MicroRNAs (miRNAs) are small non‐coding RNAs composed of 21–25 nucleotides that bind to the 3′‐untranslated region (3’‐UTR) of the target mRNA to regulate post‐transcriptional gene expression.^19^ The dysregulation of miRNAs contributes significantly to tumor development and progression.^20^, ^21^ Members of the miR‐15/107 gene family, identified by the presence of a common “AGCAGC” sequence within their seed region, have been shown to respond to a range of cellular stresses, and are implicated in various biological functions associated with cancer.^22^, ^23^, ^24^ Specifically, miR‐503, a member of the miR‐15/107 family that is specific to mammals, was found to show a more dynamic response to cellular stress than other miRNAs in the miR‐15/107 family.^22^, ^25^ In terms of function, miR‐503 has been shown to participate in the regulation of several crucial cellular processes such as proliferation, apoptosis, cell cycle, migration, and EMT.^23^, ^24^, ^26^ Notably, several studies reported that dysregulated miR‐503 is involved in tumor progression by suppressing a variety of target genes in lung cancer.^27^, ^28^, ^29^, ^30^, ^31^

In this work, we first investigated the role of miRNAs in mediating the phenotypic change of lung cancer cells in the context of acquiring resistance to targeted therapies and revealed that miR‐503 significantly dampens cell migration and invasion, EMT, and in vivo lung metastasis. Notably, we disclosed the new action of miR‐503 by identifying PTK7, an important player in collective migration, as a novel target of miR‐503. We demonstrated that miR‐503 regulates cell invasiveness by suppressing PTK7 and that PTK7 activates focal adhesion kinase (FAK), thereby driving cytoskeletal reorganization. The discovery that miR‐503 has the potential of modulating both EMT and collective migration, the two well‐characterized events in cell migration and invasion, unravels the distinguished role of miR‐503 in governing lung cancer metastasis.

## 2 MATERIALS AND METHODS

### 2.1 Cell lines

HCC827, CL1‐5, and H1299 are human lung adenocarcinoma cell lines. HCC827 cells harbor E746‐A750 deletion in the epidermal growth factor receptor (EGFR) tyrosine kinase exon 19, whereas CL1‐5 and H1299 cells express wild‐type EGFR. The HCC827 and CL1‐5 cells used in this study were provided by Dr. James Chih‐Hsin Yang and Dr. Pan‐Chyr Yang, respectively. H1299 and 293 T cells were purchased from the American Type Culture Collection (Manassas, VA). Derived gefitinib‐resistant HCC827/gef cells were generated by selection from parental HCC827 cells that have been stepwise exposed to increasing concentration of gefitinib up to 10 μM. HCC827, HCC827/gef, CL1‐5, and H1299 cells were cultured in RPMI‐1640 medium and 293 T cells were cultured in DMEM supplemented with 10% fetal bovine serum (FBS). Short tandem repeat profiling was used to authenticate cell lines. All cell lines were propagated in humidified incubators with 5% CO_2_ and at 37°C.

### 2.2 Cytotoxicity assay

Cytotoxicity assays were performed as previously described.^32^ After treatment of indicated concentrations of gefitinib for 96 h, cells were incubated with 3‐(4,5‐dimethylthiazol‐2‐yl)‐2,5‐diphenyltetrazolium bromide (MTT) to form formazan. The formazan was dissolved and the absorbance was measured at the wavelength of 550 nm with the SpectraMax® ABS Plus Microplate Reader (Molecular Devices).

### 2.3 In vitro cell migration and invasion assay

The 8 μm pore size transwell chambers were used for cell migration whereas matrigel‐coated transwell chambers were used for the invasion assay (Corning Costar Multiple Well Culture Plates, Corning, NY). Cells were suspended in serum‐free RPMI‐1640 medium and placed in the insert. The 10% FBS‐containing RPMI‐1640 medium was placed in the lower chamber. After 16  h of incubation, migratory and invasive cells were visualized and counted under a light microscope at 40‐fold magnification by staining with crystal violet solution (Sigma‐Aldrich, St. Louis, MO).

### 2.4 TaqMan® human microRNA array

The miRNA expression profiles of HCC827 and HCC827/gef cells were assessed as described previously.^33^ Briefly, extracted RNA was reverse‐transcribed and loaded onto TaqMan® array human miRNA card panels (Applied Biosystems.). The real‐time polymerase chain reaction was carried out on ABI PRISM 7900HT and analyzed using 7900RQ software (Thermo Fisher Scientific.).

### 2.5 RT‐qPCR

Quantitative real‐time PCR was performed on an ABI 7900 system according to the manufacturer's standard procedures. The TATA box‐binding protein (TBP) and RNU6B (U6B small nuclear RNA) were used as internal controls for mRNA and miRNA, respectively. The miR‐503 (#001048) and RNU6B (#001093) assay kits were from Thermo Fisher Scientific. The sequences of primers are listed in the (Table S1).

### 2.6 Western blot

Proteins were resolved by electrophoresis and immunoreactive bands for indicated proteins were visualized using chemiluminescence reagents (Millipore, Burlington, MA). The antibodies used in this study are listed in the (Table S2).

### 2.7 Phalloidin staining and immunofluorescence

Immunofluorescence staining for F‐actin, phospho‐FAK, and phospho‐paxillin was performed. Cells were seeded in chamber slides overnight, fixed with 4% paraformaldehyde, and then incubated with primary antibodies against F‐actin, phospho‐FAK, and phospho‐paxillin. The fluorescence‐conjugated F‐actin probe rhodamine phalloidin was used to stain and visualize actin filaments. Phospho‐FAK and phospho‐paxillin were visualized using a fluorescent secondary antibody (Alexa Fluor 555), and DAPI was used for staining of nuclei. Images were captured with a ZEISS LSM510 META (ZEISS, Oberkochen, Germany) and were analyzed using MetaMorph software (Nashville, TN, USA).

### 2.8 MiR‐503‐overexpressing, miR‐503‐knockdown, PTK7‐overexpressing, and PTK7‐knockdown cells

Lentivector‐based miR‐503 precursor constructs (#PMIRH503PA‐1) and pMIRNA1 (control vector) were purchased from System Biosciences (Palo Alto, CA, USA). Anti‐miR™ miR‐503 inhibitors (#AM10378) were purchased from Thermo Fisher Scientific. Human PTK7 ORF (PTK 7 open reading frame) clones (#SC118391) were purchased from Origene (Rockville, MD, USA). To establish stable miR‐503‐overexpressing cells, lentiviruses were generated and transduced into cancer cells. Transductants were isolated after 72 h of growth by using a fluorescent marker (GFP). To establish stable PTK7‐overexpressing cells, PTK7 containing the coding sequence of *PTK7* but lacking the 3′UTR was cloned into the pLKO_AS2.puro vector (National RNAi Core Facility, Academia Sinica, Taipei, Taiwan). After transduction with the pLKO_AS2.puro‐PTK7 lentivirus, the transductants were selected on puromycin‐containing media. Small hairpin RNA (shRNAs) against PTK7 (#TRCN0000006431 and #TRCN0000006435) was purchased from the National RNAi Core Facility and selected by incubating with puromycin (2 μg/mL) at a low density for 3 days; the RNA was then maintained in media containing 1 μg/mL puromycin.

### 2.9 PTK7‐3′UTR luciferase reporter assay

Wild‐type PTK7‐3′UTR reporter clone (#SC210336) and mutant PTK7‐3′UTR‐Mut reporter clone harboring six mutations in the site complementary to the seeding region of miR‐503 were purchased from Origene. For luciferase activity assay, cells were co‐transfected with miR‐503 mimic (#C‐300841‐05, Dharmacon, Lafayette, CO) in addition to wild‐type PTK7‐3′UTR or mutant PTK7‐3′UTR‐mut reporters. After 24 hours, luminescence was detected using a Dual‐Glo luciferase assay system (Promega, Madison, WI).

### 2.10 Tail vein‐based in vivo metastasis assay

All animal experiments were approved by the Institutional Animal Care and Use Committee of National Taiwan University. The lung metastasis model was conducted by injecting CL1‐5/miR‐Ctrl and CL1‐5/miR‐503 cancer cells into male NOD/SCID mice via the tail veins. After 7 weeks, the mice were sacrificed using carbon dioxide anesthesia, and lung nodules were counted under gross and microscopic examinations.

### 2.11 Statistical analysis

Data are presented as the means ± SD from at least three independent experiments. Groups were compared using the Student's t‐test. All statistical tests were two‐sided, and *p* < 0.05 was considered to be statistically significant. All analyses were conducted using the SPSS software package (version 22.0; SPSS Inc.). The Kaplan–Meier method was used to estimate the overall survival and plot the survival curve.

## 3 RESULTS

### 3.1 Lung cancer cells acquiring resistance to targeted therapy exhibit EMT and an invasive phenotype

To investigate the cellular response occurring after the acquisition of resistance to EGFR tyrosine kinase inhibitors (TKIs) in lung cancer, we generated a gefitinib‐resistant cell line using EGFR‐mutant HCC827 cells. HCC827 cells harbor an oncogenic deletion within the EGFR kinase domain (delE746‐A750 in exon 19) and are sensitive to gefitinib (IC_50_ [half maximal inhibitory concentration] <0.05 μM).

**FIGURE 1:**
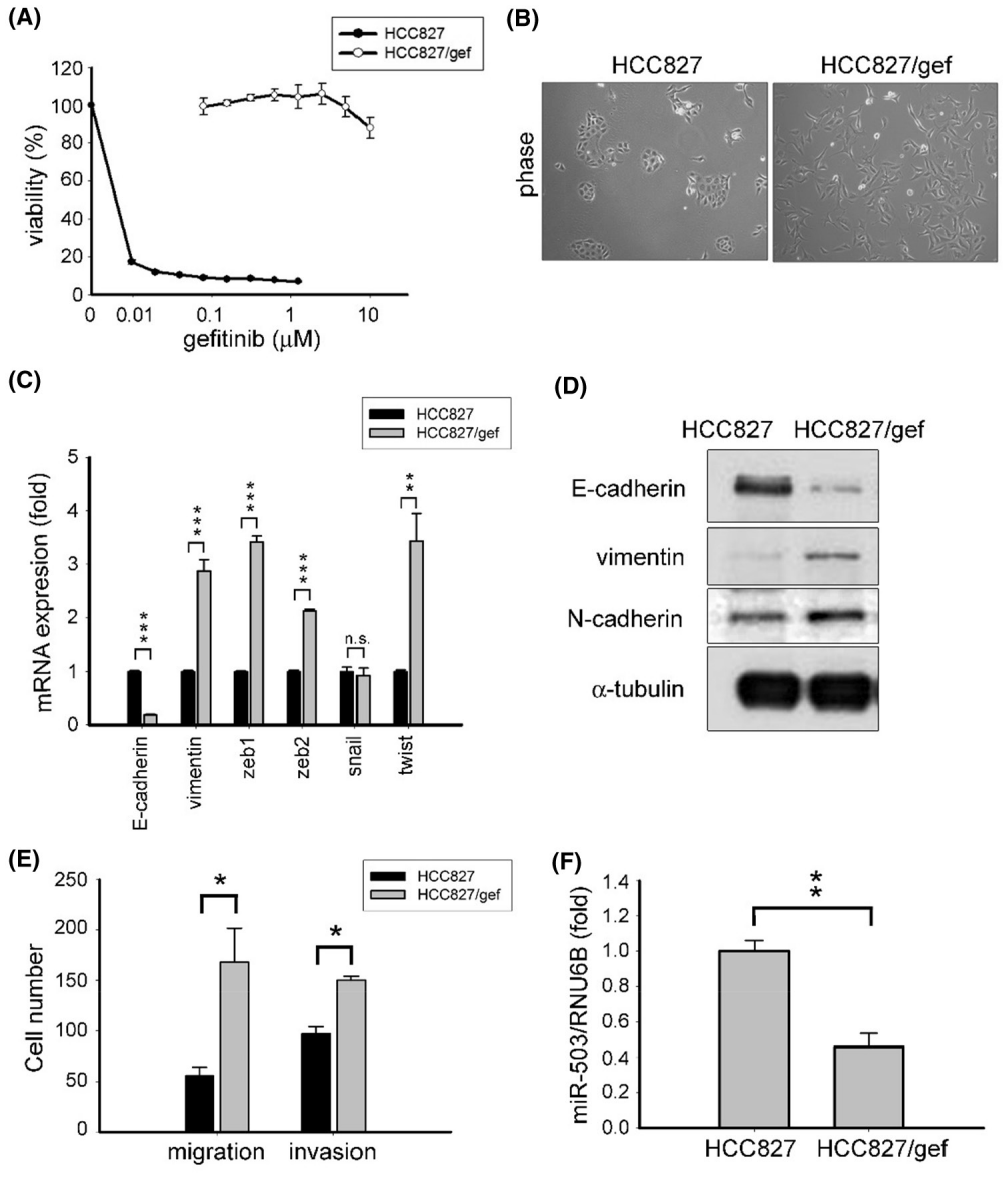
Mir‐503 is downregulated in mesenchymal‐like cells.(A) Cell viability in HCC827 and HCC827/gef cells was determined using an MTT assay. (B) Morphology of HCC827 and HCC827/gef cells evaluated using DM4000B (Leica, Wetzlar, Germany). (C) Expression of epithelial (E‐cadherin) and mesenchymal markers (vimentin) and EMT regulators (ZEB1, ZEB2, SNAIL, TWIST; **p < 0.01, ***p < 0.001). (D) Protein levels of epithelial (E‐cadherin) and mesenchymal (N‐cadherin and vimentin) markers. (E) Migratory and invasive capabilities were evaluated using transwells. Quantification of migratory and invasive cell numbers is shown herein (*p < 0.05). (F) Expression of mature miR‐503 was detected by RT‐qPCR normalized to RNU6B (**p < 0.01).

By culturing HCC827 cells in the presence of increasing concentrations of gefitinib, we isolated resistant HCC827/gef cells that were capable of growth in the presence of up to 10 μM of gefitinib. The cell viability assay confirmed an apparent difference in gefitinib sensitivity between the parental HCC827 and HCC827/gef cells (Figure 1A). As the gatekeeper EGFR T790M mutation and c‐MET (c‐mesenchymal‐epithelial transition factor) amplification are each known mechanisms of resistance to EGFR TKIs, we analyzed EGFR to check for the T790M mutation using MALDI‐TOF MS (Matrix‐Assisted Laser Desorption/Ionization Time of Flight Mass Spectrometry) genotyping analysis and quantified c‐MET gene copy number using qPCR.^34^ Although HCC827/gef cells were resistant to gefitinib, no EGFR T790M mutation or aberrant c‐MET expression was observed (Figure S1).

Next, we assessed morphological changes and EMT markers in HCC827/gef cells. Based on phase‐contrast images, HCC827/gef cells displayed a spindle‐like shape, whereas HCC827 cells exhibited epithelial morphology and increased cell‐to‐cell contacts **(**Figure 1B **)**. The RT‐qPCR analysis revealed that HCC827 cells expressed high levels of E‐cadherin, while HCC827/gef cells did not. By contrast, there was a higher level of vimentin in HCC827/gef cells compared to HCC827 cells (Figure 1C). Additionally, HCC827/gef cells showed higher mRNA expression levels of several transcription regulators associated with EMT (Figure 1C). By immunoblotting assays, a differential expression of E‐cadherin, vimentin, and N‐cadherin was observed in HCC827 cells and HCC827/gef cells (Figure 1D). To further examine the associated phenotype changes, we assessed the migratory and invasive capabilities of cancer cells using a transwell system and found that HCC827/gef cells were statistically significantly more invasive than HCC827 cells (Figure 1E). These data clearly indicate that gefitinib‐resistant HCC827/gef cells developed EMT and became more invasive than their parenteral HCC827 cells.

To explore the role of miRNAs in mediating the phenotypic change in the context of acquiring resistance to targeted therapies, we utilized the TaqMan human microRNA PCR array to screen for miRNAs that were dysregulated in HCC827/gef cells.^33^ Among the identified candidate miRNAs, we were particularly interested in miR‐503 (a miRNA in the miR‐15/107 family of miRNA genes), as members of this family have been implicated in diverse biological processes.^23^ Using an RT‐qPCR assay, we validated our hypothesis that HCC827/gef cells would show significantly lower expression of miR‐503 than their parental cells (Figure 1F), indicating that downregulation of miR‐503 was present in HCC827/gef cells acquiring resistance to EGFR TKIs. However, although we found that miR‐503 was significantly dysregulated in the derived EGFR TKI‐resistant HCC827/gef cells, ectopic overexpression of miR‐503 in HCC827/gef cells indeed did not affect drug sensitivity to EGFR TKIs, suggesting that downregulated miR‐503 did not confer drug resistance in HCC827/gef cells (Figure S2).

### 3.2 MiR‐503 suppresses EMT and reverts invasive behavior in lung cancer cells

**FIGURE 2:**
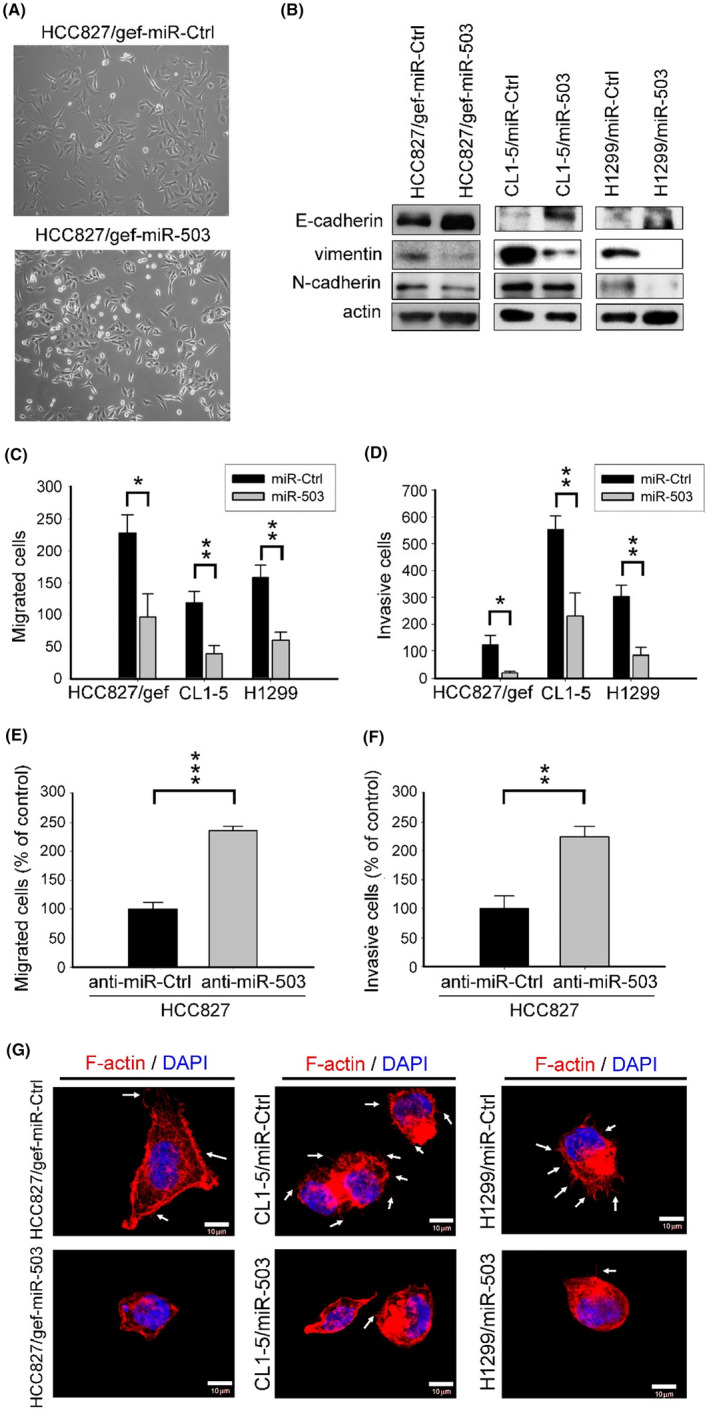
Mir‐503 suppresses cell motility.(A) Morphology of HCC827/gef‐miR‐Ctrl and HCC827/gef‐miR‐503 cells. (B) Expression of E‐cadherin, N‐cadherin, and vimentin. (C, D) Migratory and invasive capabilities in control (miR‐Ctrl) and miR‐503‐overexpressing cells. Quantification of migratory and invasive cell numbers is shown herein (*p < 0.05, **p < 0.01). (E, F) Migratory and invasive capabilities in control (anti‐miR‐Ctrl) and miR‐503‐depleted cells (anti‐miR‐503). Migratory and invasive cells were counted and normalized to the control group (anti‐miR‐Ctrl) (**p < 0.01, ***p < 0.001). (G) Rhodamine‐phalloidin staining for F‐actin (red) and nuclei (DAPI, blue) in HCC827/gef‐miR‐Ctrl and HCC827/gef‐miR‐503 cells. Linear F‐actin indicates filopodia (arrows). Scale bar = 10 μm.

We hypothesized that the downregulation of miR‐503 would mediate phenotypic changes in HCC827/gef cells. Therefore, we generated miR‐503‐overexpressing cell lines and corresponding miR‐Ctrl cell lines (miR‐Ctrl) in HCC827/gef, CL1‐5, and H1299 cells. MiR‐503 transduction markedly increased miR‐503 levels (Figure S3A,S3B). Compared to HCC827/gef‐miR‐Ctrl cells, which had a spindle‐shaped morphology, HCC827/gef‐miR‐503 cells showed a more epithelial‐like morphology (Figure 2A). Moreover, exogenous miR‐503 expression reversed EMT markers and suppressed the migratory and invasive capabilities of HCC827/gef, CL1‐5, and H1299 cells (Figure 2B,C,D). By contrast, depletion of miR‐503 expression using miR‐503‐specific inhibitors (Figure S3C) significantly increased cancer invasiveness in HCC827 cells (Figure 2E,F). These data indicate that miR‐503 restrained EMT and invasive behavior in lung cancer cells.

Cancer cells reorganize their cortical actin cytoskeletons to enable dynamic elongation and directional motility. Actin‐rich membrane projections, such as lamellipodia and filopodia, facilitate cell movement. Given the pronounced effect of miR‐503 on altering cell morphology and slowing down cell migration, we aimed to evaluate whether miR‐503 modulates cytoskeletal dynamics. Dynamic changes in the actin cytoskeleton in HCC827/gef, CL1‐5, and H1299 cells, with or without exogenous miR‐503 expression, were assessed using phalloidin staining for F‐actin as well as through imaging by confocal microscopy. Strikingly, cells overexpressing miR‐503 showed a more round‐shaped morphology and displayed a marked decrease in filopodia as compared to their control cells (Figure 2G). These results indicate that miR‐503 regulates cytoskeletal dynamics in lung cancer cells.

### 3.3 Wnt/planar cell polarity protein PTK7 is the direct target of miR‐503

**FIGURE 3:**
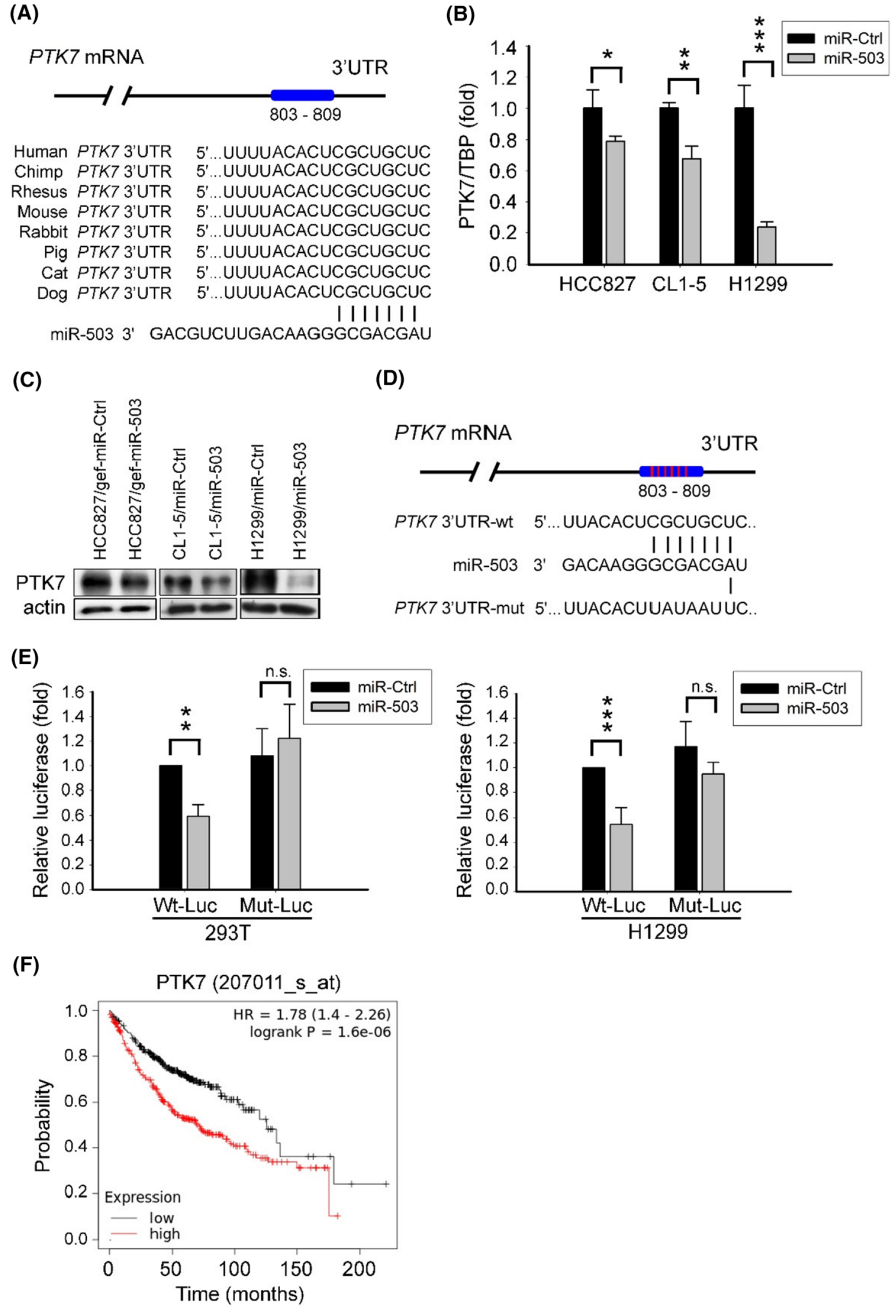
(A) Mir‐503 targets the 3′UTR of PTK7 mRNA.The putative miR‐503 binding site (CGCUGCU) in the 3′UTR in human PTK7 (nt 803 ~ 809) mRNA. (B, C) Expression levels of PTK7 mRNA and protein in miR‐Ctrl or miR‐503‐overexpressing cells (*p < 0.05, **p < 0.01, ***p < 0.001). (D) Schematic representation of the mutant sequences (UAUAAUU) on the seeding region of the miR‐503 binding site. The sequences encoding wild‐type and mutated fragments of the PTK7 3′UTR are depicted herein. (E) miR‐503 suppressed PTK7 via direct interaction with its 3′UTR. 293 T or H1299 cells were co‐transfected with pMir‐PTK7‐3′UTR‐wt or pMir‐PTK7‐3′UTR‐mut reporter plasmids, in addition to miR‐Ctrl (scrambled control) or miR‐503 mimics. The relative luciferase activities were normalized to renilla (**p < 0.01, ***p < 0.001). (F) Overall survival analysis with respect to PTK7 mRNA expression levels in patients with lung adenocarcinoma (*n* = 719; log‐rank *p* < 0.001) using the Kaplan–Meier Plotter database (http://kmplot.com/analysis/).

To understand the biological effects of miR‐503 in lung cancer cells, we used the TargetScan prediction algorithm (https://www.targetscan.org/) to search for putative miR‐503 target genes. We identified PTK7, a Wnt/PCP protein, as one of the most promising candidates. Sequence alignment showed that PTK7 carries a predicted 7‐mer miR‐503 binding sequence (CGCUGCU) in its 3′UTR region that is highly conserved across mammalian species (Figure 3A). We found that ectopic expression of miR‐503 resulted in decreased levels of PTK7 mRNA and protein in different lung cancer cells (Figure 3B,C). Using the luciferase reporter assay, we further demonstrated that miR‐503 binds to the 3′UTR of PTK7 mRNA in 293 T cells and H1299 cells (Figure 3D,E). This binding was abrogated by the introduction of mutations restricted to the target sequence (UAUAAUU) in the 3′UTR region of PTK7 (Figure 3D,E).

Collectively, these data indicate that the Wnt/PCP protein PTK7 is the direct target of miR‐503. Finally, using the Kaplan–Meier Plotter database (http://kmplot.com/analysis/), we disclosed that higher levels of PTK7 mRNA were statistically significantly associated with poorer overall survival in patients with lung adenocarcinoma (*n* = 719; log‐rank *p* < 0.001) (Figure 3F), thereby implicating PTK7 in lung cancer progression.

### 3.4 PTK7 activates FAK and paxillin but not EMT

**FIGURE 4:**
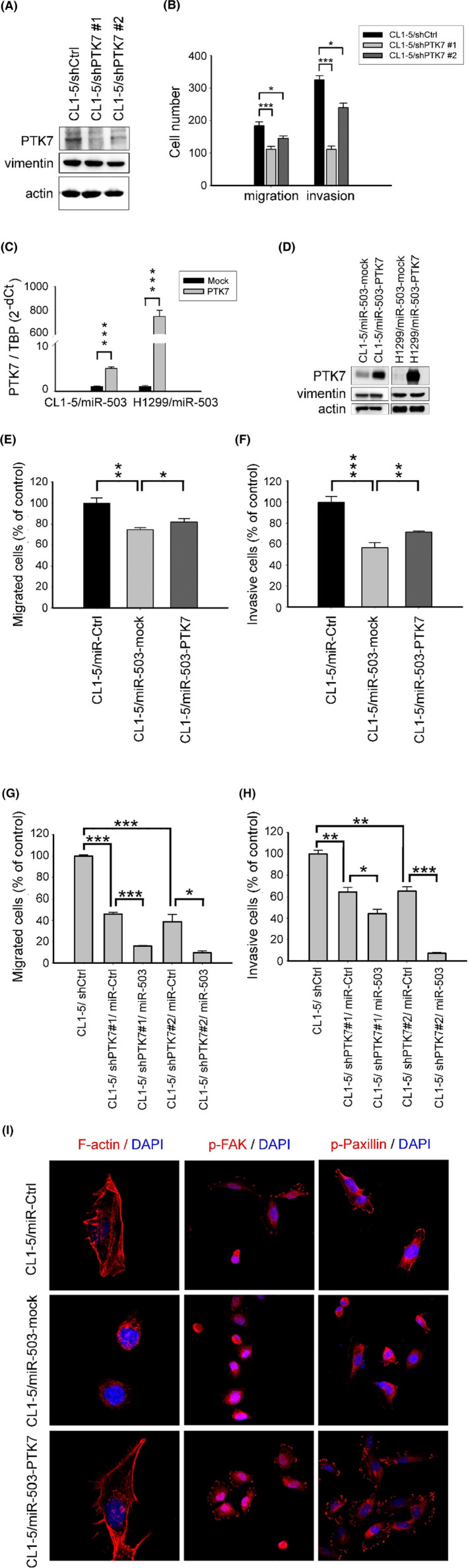
Restoration of PTK7 reversed miR‐503‐mediated repression of cell migration and invasion.(A) Immunoblotting was conducted to determine the expression levels of PTK7 and vimentin proteins. (B) Migratory and invasive capabilities in control (shCtrl) and PTK7‐knockdown (shPTK7) cells. Quantification of migratory and invasive cell numbers is shown herein (*p < 0.05, ***p < 0.001). (C, D) CL1‐5/miR‐503 and H1299/miR‐503 cells stably expressing PTK7 were established by infecting miR‐503‐overexpressing cells with PTK7 lentivirus particles containing the PTK7‐encoding sequence but lacking the 3′UTR, and PTK7 expression was determined by RT‐qPCR and immunoblotting (***p < 0.001). (E, F) Migratory and invasive capabilities in control (mock) and PTK7‐overexpressing cells. Migratory and invasive cells were counted and normalized to the control group (CL1‐5/miR‐Ctrl) (*p < 0.05, **p < 0.01, ***p < 0.001). (G, H) Migratory and invasive capabilities in PTK7‐knockdown (shPTK7) cells overexpressing with control or miR‐503. Migratory and invasive cells were counted and normalized to the control group (CL1‐5/shCtrl) (*p < 0.05, **p < 0.01, ***p < 0.001). (I) Merged photomicrographs of rhodamine‐phalloidin staining for F‐actin (red), phospho‐FAK (red), phospho‐paxillin (red), and nuclei (DAPI, blue) in mock and PTK7‐overexpressing cells. Magnification is 1000x for F‐actin and 400x for phospho‐FAK and phospho‐paxillin.

To further explore the function of PTK7, we used shRNA to knock down PTK7 expression in CL1‐5 cells (Figure 4A). Our data revealed that downregulation of PTK7 represses the migratory and invasive capabilities of CL1‐5 cells (Figure 4B). However, the protein level of vimentin was not altered **(**Figure 4A **)**.

To test whether miR‐503 exerts its effects by inhibiting PTK7, PTK7‐overexpressing cells (CL1‐5/miR‐503‐PTK7 and H1299/miR‐503‐PTK7) were generated by transducing the cDNA of PTK7 without the 3′UTR. Successful overexpression of PTK7 was verified at both the mRNA and protein levels (Figure 4C,D). Because miR‐503 prominently modulates EMT, we evaluated whether PTK7 coordinates this process. However, we found that reconstitution of PTK7 expression did not affect the protein levels of vimentin or the mRNA expression of E‐cadherin, vimentin, or N‐cadherin in both CL1‐5 and H1299 cells (Figure 4D and Figure S4). According to these findings, it was suggested that PTK7 is not involved in miR‐503‐induced EMT and that miR‐503 suppresses EMT by inhibiting other undetermined target genes.

Although PTK7 did not influence EMT, we found that reconstitution of PTK7 expression restored the migratory and invasive capabilities of CL1‐5 and H1299 cells overexpressing miR‐503 (Figure 4E,F and Figure S5), indicating that PTK7 was the direct target gene that mediates the function of miR‐503 in controlling cancer invasiveness. On the other hand, we wondered whether miR‐503 regulates cancer invasiveness only through the target inhibition of PTK7. Using shRNA to deplete PTK7 expression in CL1‐5 cells, followed by ectopic overexpression of miR‐503 in these cells (CL1‐5/shPTK7#1/miR‐503 and CL1‐5/shPTK7#2/miR‐503 cells), we evaluated the migratory and invasive capabilities of these cells compared with those transfected with corresponding controls (CL1‐5/shPTK7#1/miR‐Ctrl and CL1‐5/shPTK7#2/miR‐Ctrl). Specifically, our data revealed that in the PTK7‐knockdown CL1‐5 cells, cancer cells with exogenous overexpression of miR‐503 still exhibited less invasive phenotypes (Figure 4G,H). These data suggested that miR503, in addition to interacting with PTK7, pleiotropically controls cancer cell invasiveness through modulating other undetermined target genes and/or signaling programs.

Importantly, while we observed loss of filopodia and round‐shaped morphology in cells overexpressing miR‐503, reconstitution of PTK7 expression resulted in an obvious increase in filopodia, suggesting that PTK7 reversed the effect of miR‐503 on actin dynamics (Figure 4I). These findings indicate that PTK7 acts as an effector of miR‐503 and promotes cell motility by regulating cytoskeletal dynamics independently of EMT. FAK is the most prominent protein tyrosine kinase involved in regulating actin cytoskeleton dynamics. Activation of FAK and its downstream adaptor protein paxillin by phosphorylation is a hallmark of cytoskeletal remodeling and cell migration. While miR‐503 overexpression suppressed FAK and paxillin phosphorylation in CL1‐5 and H1299 cells, reconstitution of PTK7 expression facilitated FAK and paxillin activation (Figure 4I).

### 3.5 MiR‐503 inhibits PTK7 expression and suppresses metastasis in vivo

**FIGURE 5:**
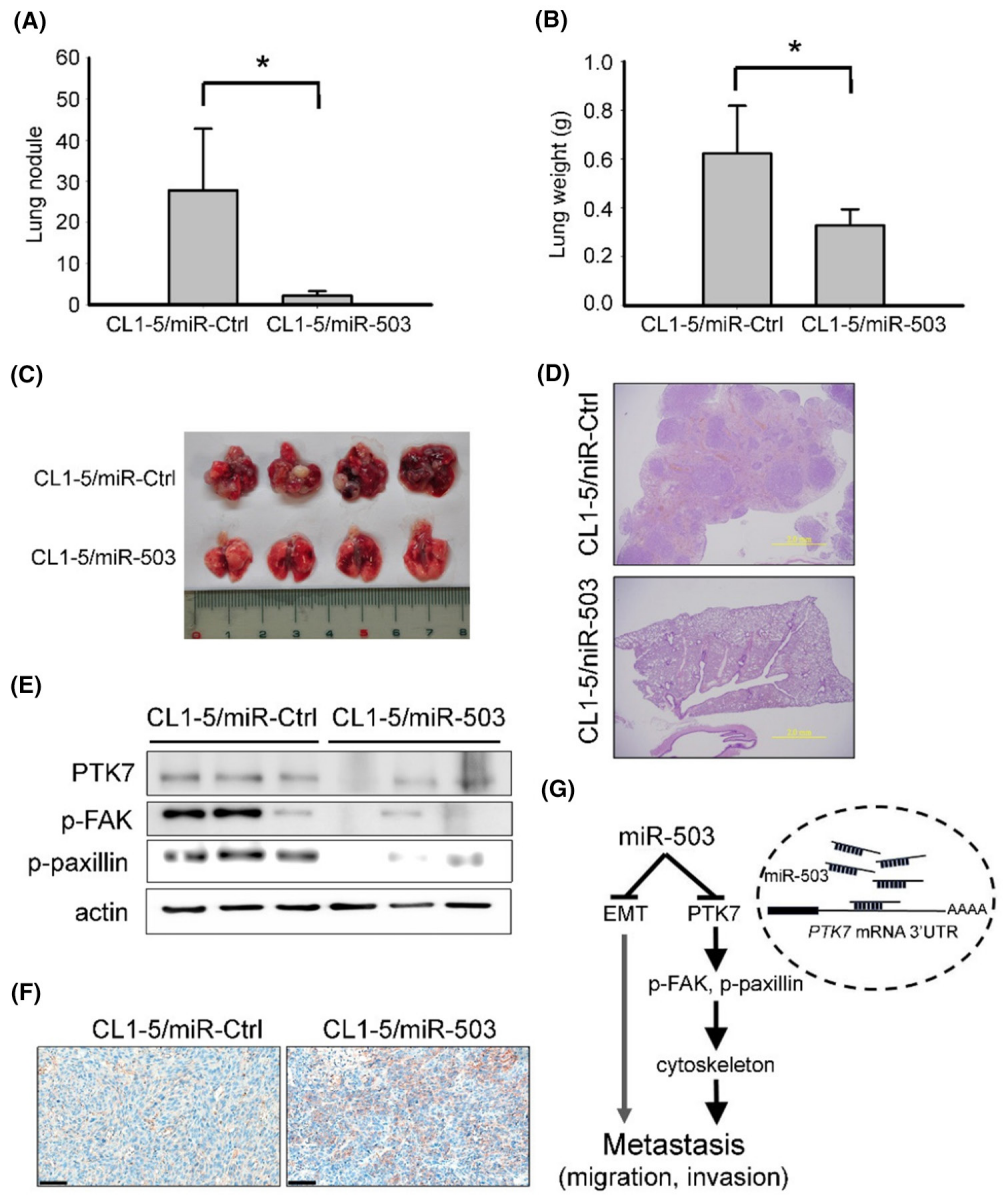
Overexpression of miR‐503 inhibited lung metastasis in vivo.(A–D) Mir‐503 inhibited lung metastasis in NOD/SCID mice. We calculated the number of lung metastasis nodules (A) as well as weights (B) (*n* = 5). Lung metastasis (C) and HE (hematoxylin and eosin) staining (D) of the respective tissues from NOD/SCID mice are shown herein. Scale bar = 2 mm. (E) Protein expression in representative lung tissues. (F) Immunohistochemistry staining of membranous E‐cadherin in representative tumor tissues. Scale bar = 100 μm. (G) Schematic diagram showing the role of miR‐503 in regulating cancer metastasis.

To recapitulate the in vitro findings and examine the in vivo effect of miR‐503 in controlling metastatic capability, we injected miR‐503‐overexpressing and corresponding miR‐Ctrl cell lines (CL1‐5/miR‐Ctrl and CL1‐5/miR‐503) into the lateral tail veins of NOD/SCID mice. All mice were planned to be sacrificed 7 weeks later and their lungs were excised for further examination. However, there were two mice (one in each group) that were quite ill‐looking just several days before the scheduled time of sacrifice and were thus sacrificed earlier. The excised lungs from these two mice were included in the measurements of the lung nodules and lung weights. Our results revealed that miR‐503‐expressing cancer cells formed fewer and smaller metastatic lesions on the surface of the lungs (Figure 5A,B,C). In each of the evaluated groups, lung metastases were confirmed by histological examination of the dissected lungs (Figure 5D). Consistent with the data from cultured cells, immunoblotting assays demonstrated the regulatory effect of miR‐503 on PTK7, FAK, and paxillin in mouse tumor tissues (Figure 5E). Furthermore, immunohistochemistry assays revealed increased expression of membranous E‐cadherin in tumor cells overexpressing miR‐503 (Figure 5F). Collectively, these data provide in vivo evidence supporting the important role of dysregulated miR‐503 in lung cancer metastasis. Mechanistically, our findings suggest that miR‐503 simultaneously suppresses EMT and PTK7 expression. Through the post‐transcriptional control of PTK7 expression, miR‐503 regulates the activation of FAK and paxillin, which are involved in cytoskeletal remodeling (Figure 5G).

## 4 DISCUSSION

Lung cancer has a high potential for metastasis. Even in early‐stage lung cancer, vascular invasion is often seen in resected lung tumors.^35^ According to the data collected by the Surveillance, Epidemiology, and End Result (SEER) program of the US National Cancer Institute (NCI) for the years 1988–2001, 40% of lung cancer patients had detectable distant metastases at diagnosis.^36^ In the clinical setting, metastatic spread is the major cause of treatment failure and death in patients with lung cancer. Examining new paradigms of lung cancer invasion and metastasis, such as the role of miRNAs, is therefore crucial for uncovering therapeutic opportunities,

There is strong evidence to support that miR‐503 acts as a tumor suppressor since its downregulation contributes to cancer progression.^23^, ^24^ In recent years, several studies have revealed the functional roles of miR‐503 in lung cancer. For example, Wang et al. reported that miR‐503 inhibits cell proliferation by targeting cyclin D1, and Qui et al. showed that miR‐503 regulates apoptosis by targeting Bcl‐2 (B‐cell lymphoma 2) in lung cancer.^27^, ^28^ Furthermore, Yang et al. revealed that miR‐503 directly inhibits PI3K (phosphatidylinositol 3‐kinase) p85 and IKK‐β (nuclear factor kappa B kinase subunit beta) to suppress proliferation, migration, and invasion in lung cancer cells. In addition to providing in vivo evidence that miR‐503 retrains tumor growth and metastasis, they showed that miR‐503 is a prognostic marker in patients with lung cancer.^29^ Other studies have shown that miR‐503 inversely regulates lung cancer progression by inhibiting different targets including PDK1 (pyruvate dehydrogenase kinase 1) and LARP1 (La‐related protein 1) respectively.^30^, ^31^ In this study, we disclosed the unknown action of miR‐503 to modulate lung cancer metastasis. We demonstrated that miR‐503 not only regulates EMT but also inhibits migration and invasion by targeting PTK7 in lung cancer cells. It is increasingly known that cancer invasion and metastasis fundamentally rely on collective migration and that PTK7 is required in this process.^9^, ^10^, ^11^, ^12^, ^13^ Through the direct inhibition of PTK7 expression, miR‐503 is capable of participating in the regulation of collective migration. By contrast, the significance of EMT during cancer dissemination remains debatable. However, it has been widely accepted that EMT is triggered in invasive cancer cells. As a result of EMT, cells lose their polarity, cell–cell junctions, and adherence to the basement membrane, and are able to migrate and invade as a single cell.^5^ Additionally, EMT may provide survival advantages and immune evasion, thereby increasing the spread of cancer cells.^5^ Notably, the observations that cancer cells with partial or hybrid EMT can move jointly, as well as that leading cells in collective migration undergo EMT, indicated that these EMT and collective migration may be interrelated in cancer metastasis.^14^, ^15^, ^16^, ^17^, ^18^ Our study strikingly unraveled the pleiotropic regulatory functions of miR‐503 in controlling lung cancer metastasis. Accordingly, miR‐503 may represent a promising therapeutic target in lung cancer metastasis to kill two birds with one stone.

It has been shown that miR‐503 expression is prominently altered by cellular stress.^23^, ^25^ In this study, we found that miR‐503 is significantly downregulated in response to targeted therapy. Moreover, dysregulated miR‐503 confers aggressive behavior in lung cancer cells. These results are consistent with the idea that the stress response induced by anticancer treatments can enhance the ability of cancer cells to spread.^37^ Indeed, there is an emerging notion that therapeutic modalities can promote metastasis by disrupting tumor cells or their environments.^38^, ^39^, ^40^ As far as we know, there is no strong clinical evidence that targeted therapies facilitate the dissemination of lung cancer cells. However, preclinical cancer models have demonstrated that targeted therapies can promote metastasis. For instance, in an animal study of melanoma, treatment with vemurafenib resulted in increased metastasis of vemurafenib‐resistant tumors in the model mice.^41^ According to these observations, as well as the finding that dysregulated miR‐503 promotes lung cancer dissemination in our study, we speculate that miR‐503 may play an important role in mediating cancer dissemination induced by targeted therapies in lung cancer.

Consistent with previous reports, this study showed that miR‐503 suppresses EMT.^23^, ^42^, ^43^ The mechanism underpinning the regulation of EMT by miR‐503 in lung cancer, however, was not determined. Various studies have reported that miR‐503 can target SMURF1 and SMURF2 (SMAD ubiquitination regulation factors 1 and 2), thereby enhancing TGF‐β (transforming growth factor beta) signaling in individual biological processes other than cancer.^42^, ^43^ These observations certainly imply that miR‐503 may contribute to TGF‐β‐induced EMT under certain circumstances. However, miR‐503 target selection may be specific to the individual context. In lung cancer cells, it has been reported that miR‐503 targets PI3K p85, IKK‐β, and PDK1 to suppress invasive characteristics.^29^, ^30^ However, whether the invasive traits were caused by EMT and thus whether miR‐503 inhibits EMT by suppressing these targets have not been investigated. Given that miR‐503 regulates EMT and that PTK7 was identified as a novel target of miR‐503, we considered it of special importance to examine whether miR‐503 suppresses EMT by inhibiting PTK7 expression. However, we found that PTK7 had no noticeable impact on vimentin levels in lung cancer cells. Coupled with the results of a previous study, which showed that E‐cadherin levels were not altered in PTK7 knockdown cells, we excluded a causal link between PTK7 and miR‐503‐induced EMT.^44^ Instead, our results suggest that miR‐503 regulates PTK7 expression and EMT in parallel in lung cancer cells. Further studies are needed to explore the underlying mechanism through which miR‐503 regulates EMT in lung cancer cells.

PTK7 was identified to be a direct target of miR‐503 in this study. Additionally, re‐expressing PTK7 restored the functional effects of miR‐503, indicating that miR‐503 restrains cell invasiveness by inhibiting PTK7. As an evolutionarily conserved pseudo‐kinase, PTK7 functions as a Wnt co‐receptor in the non‐canonical Wnt/PCP signaling pathway.^45^ It is well‐known that the non‐canonical Wnt/PCP pathway controls the orientation of epithelial cells within a tissue (i.e., planar polarity) as well as directional motility.^46^, ^47^, ^48^ Studies using model systems have clearly confirmed that PTK7 is required for morphogenetic cell movements during embryonic development, such as neural crest migration and convergent extension during gastrulation.^9^, ^10^, ^11^ Furthermore, PTK7 is one of several cell surface molecules involved in contact inhibition of locomotion (CIL), referring to the stoppage of cell movement and repolarization of the migration machinery on cell–cell collisions during collective migration.^12^, ^49^ Together, these observations suggest that PTK7 is an important polarity‐determining protein that plays a substantial role in collective cell migration. Accordingly, we propose that, through its inhibitory effect on PTK7, miR‐503 has the potential of participating in the modulation of collective migration.

PTK7 is upregulated in many types of cancers including lung cancer.^50^ According to the Kaplan–Meier Plotter database (http://kmplot.com/analysis/), we showed that PTK7 expression was significantly associated with survival in patients with lung cancer, suggesting that PTK7 is involved in cancer progression. Although PTK7 is well known to be required in collective migration, its functional roles in promoting cancer progression have not been well elucidated. After finding that PTK7 enhances cell migration, invasion, and cytoskeletal reorganization in this study, we focused on the focal adhesion signaling pathway to explore the mechanism by which PTK7 regulates cell motility. The signaling events at focal adhesions can reorganize the actin cytoskeleton, change the cell shape, and increase motility.^51^ Specifically, it has been established that FAK is a key mediator of focal adhesion signaling.^52^ In the signaling pathway, activated FAK recruits Src to form the FAK–Src signaling complex, which phosphorylates other adapter and signal proteins, such as paxillin, to regulate the cytoskeletal organization and directional cell migration.^52^ Our study demonstrated that PTK7 activates FAK and paxillin, and facilitates the formation of filopodia, which are necessary for cell migration. Although the role of PTK7 in polarity establishment and coordinated cell movements is well known, our findings linking PTK7 to the focal adhesion signaling pathway to regulate cytoskeletal reorganization shed more light on the function of PTK7 to promote cancer progression.

There are limitations to this study. First, whereas our results revealed that miR‐503 inhibits EMT, the target gene of miR‐503 that mediates the effect of miR‐503 in regulating EMT was not identified. In addition, based on the fact that PTK7 is pivotal to cohesive migration, as well as our results that PTK7 facilitates migratory and invasive capacities of lung cancer cells, we propose that miR‐503 might modulate collective migration and invasion by inhibiting PTK7. However, the matrix invasion assays used in this study might not offer a satisfactory approach to examining collective migration and invasion. In recent years, studies have used multicellular tumor spheroids or organoids for the evaluation of collective migration and invasion.^53^ Finally, the tail‐vein injection model, rather than the orthotopic tumor model, was used to assess the in vivo effect of miR‐503 in suppressing lung metastasis in our study. Certainly, the orthotopic model has the advantage of mimicking spontaneous metastasis. However, by tail‐vein injection model, we have demonstrated the significant role of miR‐503 in controlling in vivo lung metastasis.

## 5 CONCLUSIONS

In the current study. we disclosed the novel roles of miR‐503 and PTK7 in governing lung cancer metastasis. MiR‐503 not only suppresses EMT but also targets PTK7 to inhibit invasiveness in lung cancer cells. These findings remarkably implied that miR‐503 potentially orchestrates both EMT and collective migration, the two distinguished mechanisms in cancer metastasis. Furthermore, we unraveled that PTK7 facilitates cell motility by activating FAK, thereby controlling cytoskeletal reorganization. Collectively, we propose that miR‐503 represents a pleiotropic regulator of cancer dissemination and hence an attractive therapeutic target for lung cancer metastasis.

## AUTHOR CONTRIBUTIONS

**Tzu Hsiu Tsai:** Conceptualization (equal); funding acquisition (equal); investigation (equal); writing – original draft (equal); writing – review and editing (equal). **Chien‐Hung Gow:** Conceptualization (equal); funding acquisition (equal); writing – original draft (equal). **Yi‐Nan Liu:** Conceptualization (equal); investigation (equal); writing – original draft (equal). **Meng‐Feng Tsai:** Conceptualization (equal); supervision (equal). **Tzu‐Hua Chang:** Investigation (equal). **Shang‐Gin Wu:** Investigation (equal). **Min‐Shu Hsieh:** Investigation (equal). **Kang‐Yi Su:** Investigation (equal). **Jin‐Yuan Shih:** Conceptualization (equal); funding acquisition (equal); supervision (equal); writing – original draft (equal); writing – review and editing (equal).

## FUNDING INFORMATION

The study was supported by grants from the Ministry of Science and Technology (grant numbers 101‐2314‐B‐002‐174, 102‐2314‐B‐002‐098, and 103‐2314‐B‐002‐143) and the Far Eastern Memorial Hospital‐National Taiwan University Hospital Joint Research Program grant (grant number 108‐FTN15), Taiwan.

## CONFLICT OF INTEREST STATEMENT

J‐Y Shih has served as an advisory board member from Amgen, AstraZeneca, Boehringer Ingelheim, Bristol‐Myers Squibb Pfizer, CStone Pharmaceuticals, Novartis, Merck Sharp & Dohme, Ono Pharmaceutical, Takeda, and Janssen; received speaking honoraria from ACTgenomics, Amgen, AstraZeneca, Boehringer Ingelheim, Bristol‐Myers Squibb, Genconn Biotech, Roche, Bayer, Eli Lilly, Pfizer, Novartis, Merck Sharp & Dohme, CStone Pharmaceuticals, Chugai Pharma, Takeda, Janssen, TTY Biopharm, Orient EuroPharma, and MundiPharma; as well as a grant from Roche. There are no conflicts of interest among other authors.

## ETHICS STATEMENT

The National Taiwan University Institutional Animal Care and Use Committee approved all animal experiments, which followed all relevant national and international guidelines (such as ARRIVE).

## Supporting information


Data S1.

## Data Availability

The data used for this study, though not available in a public repository, will be made available to other researchers upon reasonable request.
