# Supplementary material for: MiR‐503 pleiotropically regulates epithelial‐mesenchymal transition and targets PTK7 to control lung cancer metastasis
Source: Cancer Med. 2023 May 22;12(13):14511–25. doi: 10.1002/cam4.6116 (PMC10358207; doi:10.1002/cam4.6116)
Supplement: Supplementary file 1 — Data S1. [file CAM4-12-14511-s001.docx]

**SUPPORTING INFORMATION**

**Supplementary Figures**

**
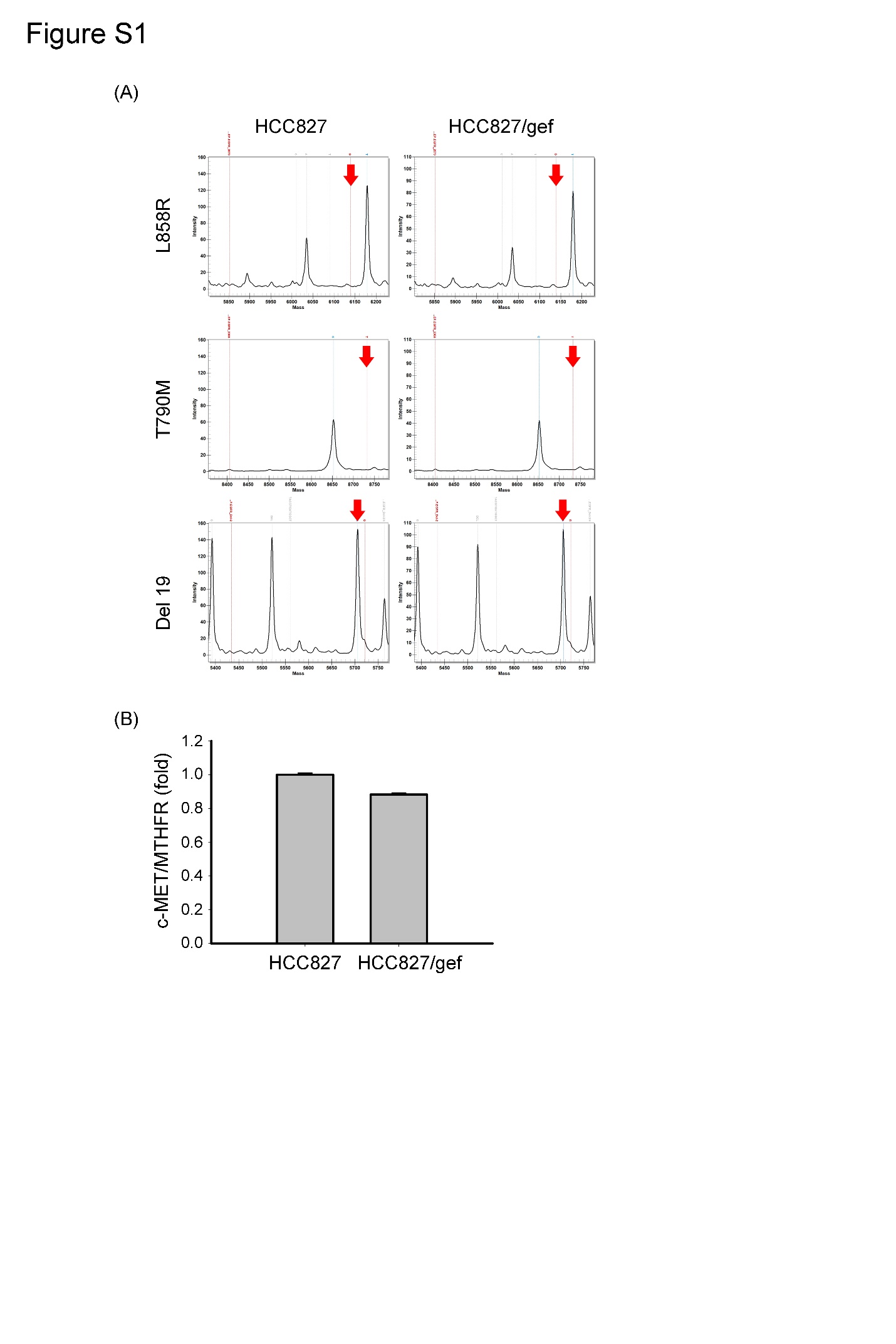
**

**Figure S1** A lack of T790M mutation or MET amplification in HCC827/gef cells.

**(A)** EGFR T790M detection (i.e., a C to T alteration in the 2,369th nucleotide) using highly sensitive MALDI-TOF nucleotide mass spectrometry. Shifted signals due to the incorporated nucleotides (C or T) of unextended detection probes (U) following a single nucleotide extension reaction can be identified in the spectrum. HCC827 cells harbor exon 19 deletions. No T790M mutation was detected in HCC827/gef cells. **(B)** MET copy number was evaluated by RT-qPCR (real-time quantitative polymerase chain reaction) within genomic DNA. No MET amplification was noted in HCC827/gef cells. The represented values indicate the mean of triplicate evaluations for each cell.

**
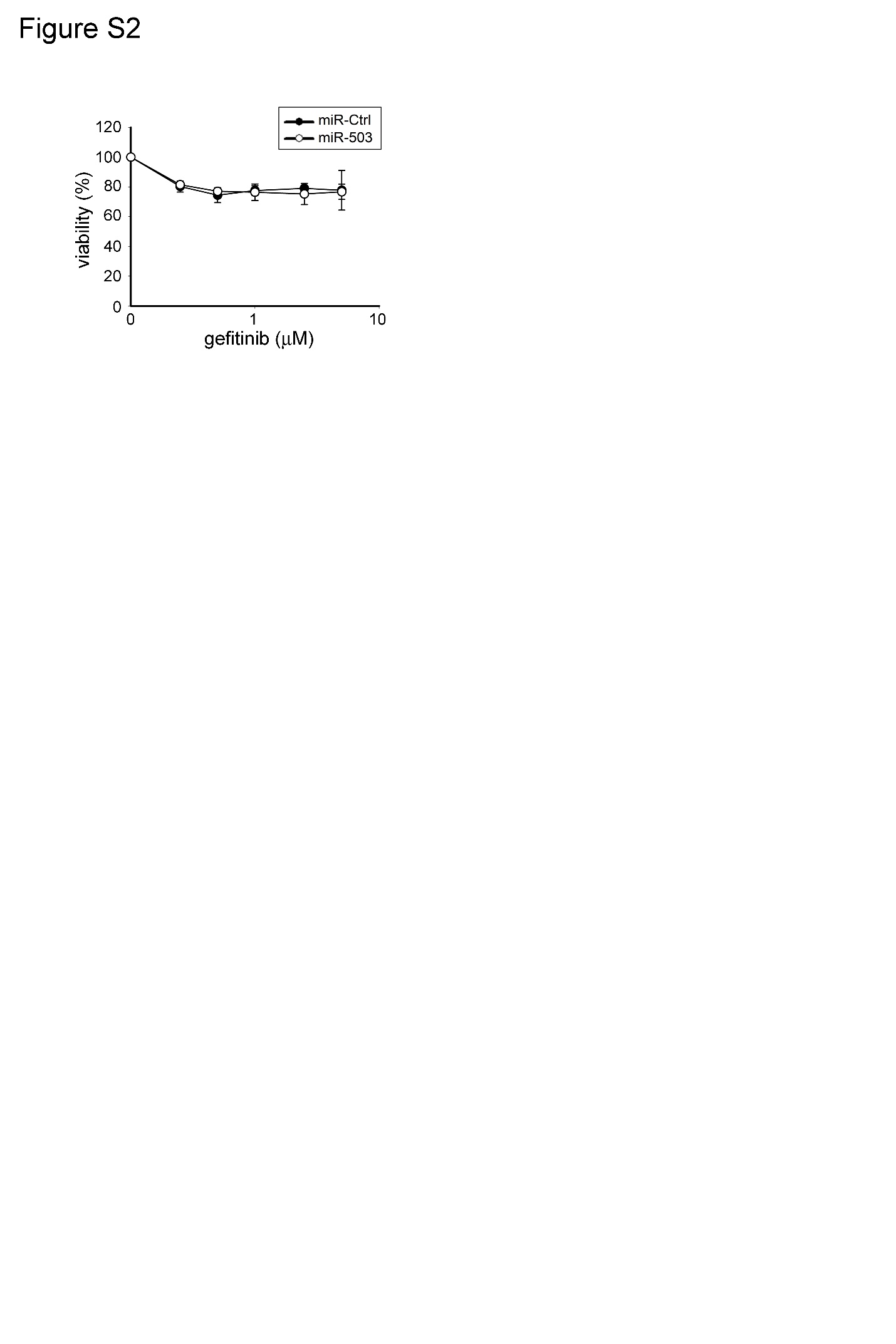
**

**Figure S2** Sensitivity to gefitinib in control and miR-503-overexpressing HCC827/gef cells.

Drug sensitivity to gefitinib in HCC827/gef cells with control (miR-Ctrl) or miR-503 overexpression was determined using an MTT assay. Although miR-503 was significantly dysregulated in the derived EGFR TKI-resistant HCC827/gef cells, ectopic overexpression of miR-503 in HCC827/gef cells did not affect the sensitivity to gefitinib.

**
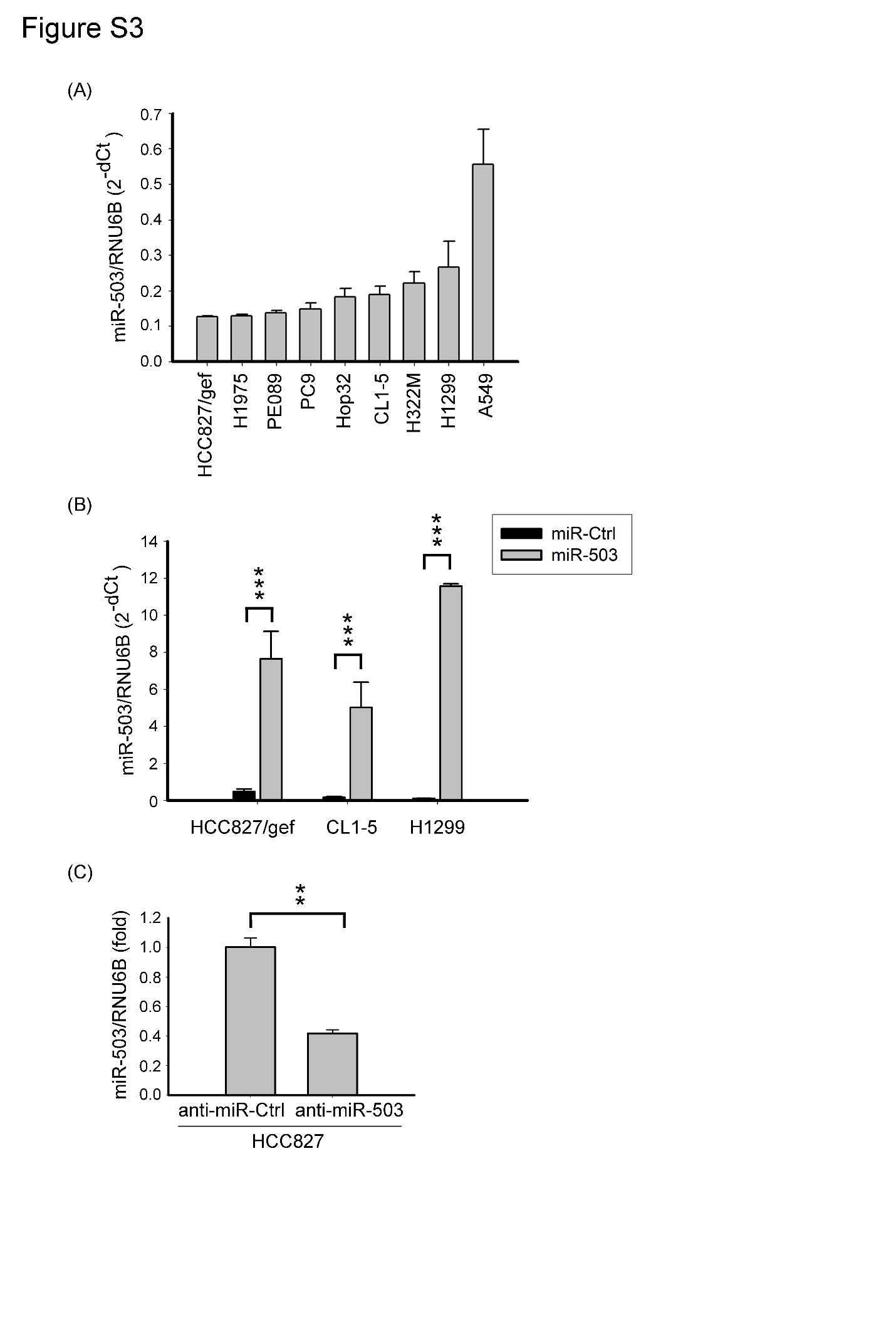
**

**Figure S3** MiR-503 expression levels in indicated lung cancer cells.

MiR-503 expression was measured using RT-qPCR (real-time quantitative polymerase chain reaction), and normalized to RNU6B (U6B small nuclear RNA) expression in **(A)** a variety of lung cancer cell lines, **(B)** control or miR-503-overexpressing lung cancer cells, and **(C)** HCC827 cells with control (anti-miR-Ctrl) or miR-503 knockdown using miR-503-specific inhibitors (***p* <0.01, ****p* <0.001).

**
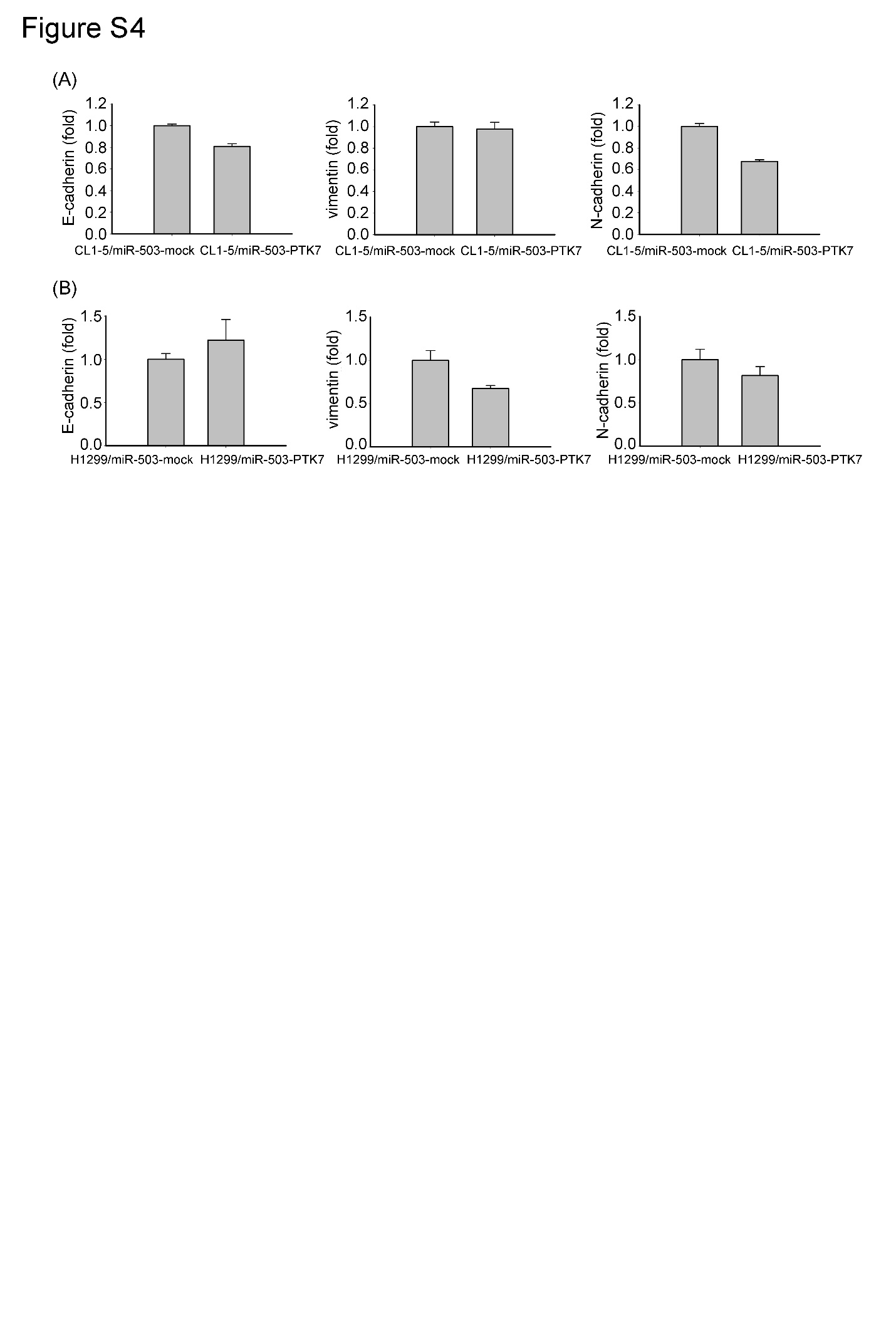
**

**Figure S4** RNA levels of EMT markers in control and PTK7-overexpressing cells.

RT-qPCR (real-time quantitative polymerase chain reaction) was conducted to determine expression levels of E-cadherin, N-cadherin, and vimentin in control (mock) or PTK7-overexpressing CL1-5/miR-503 **(A)**, as well as H1299/miR-503 **(B)** cells.

**
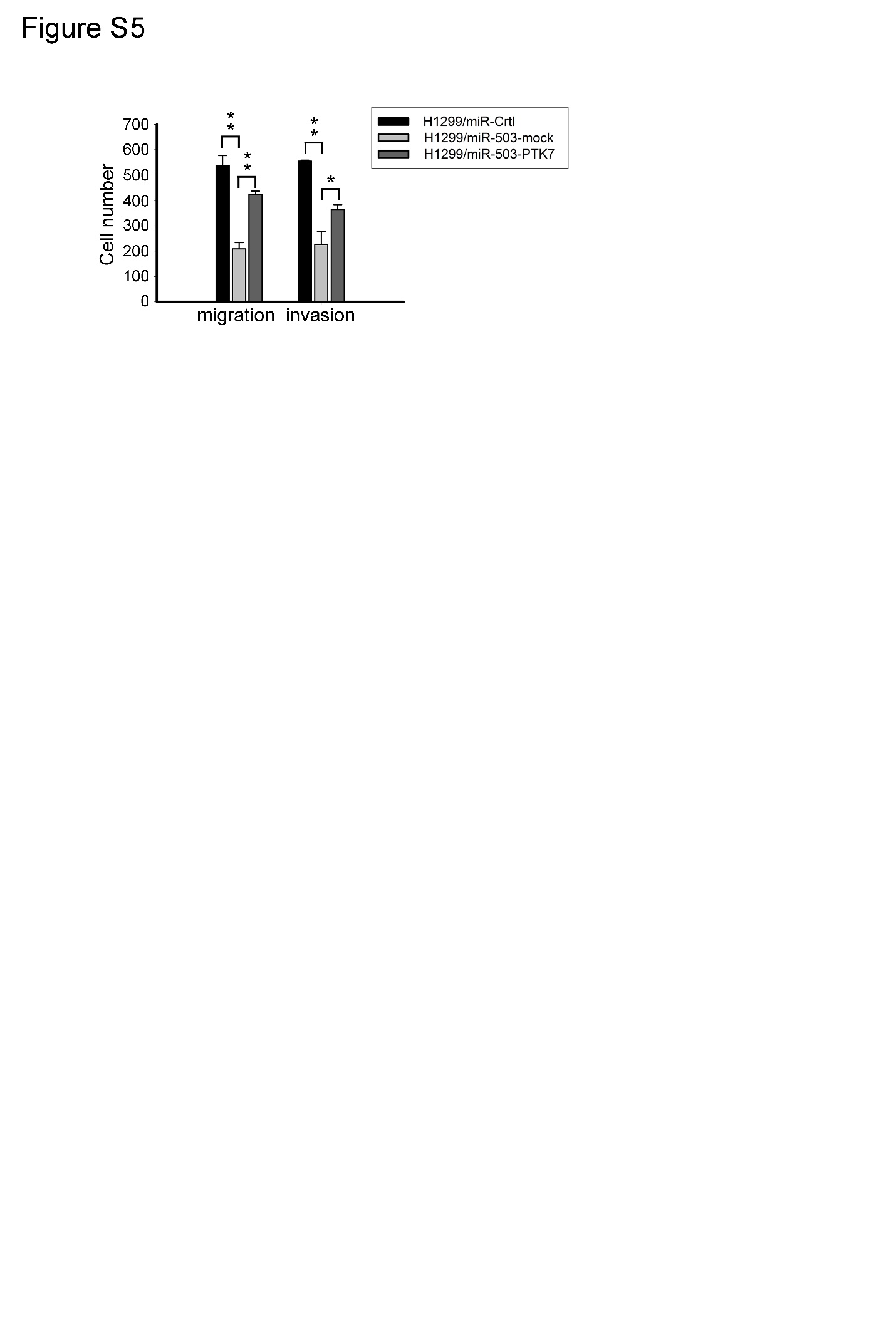
**

**Figure S5** Migratory and invasive capabilities in control and PTK7-overexpressing cells.

Migratory and invasive capabilities in control (mock) and PTK7-overexpressing H1299/miR-503 cells were evaluated using a transwell system. Quantification of migratory and invasive cell numbers is shown herein (**p* <0.05, ***p* <0.01).

**Supplementary Tables**

**Table S1** Primers for quantitative real-time PCR used in this study.

| **Primers for real-time PCR used in this study** | | |
| --- | --- | --- |
| Gene name | forward | reverse |
| E-cadherin (SYBR) | 5'-CCGAGAGAGTTTCCCTACGTATACC-3' | 5'-CCCTTGTACGTGGTGGGATT-3' |
| N-cadherin (SYBR) | 5'-GTCAGTGAAGGAGTCAGCAGAAGTT-3' | 5'-AGTCTCTCTTCTGCCTTTGTAGGTG-3' |
| vimentin (SYBR) | 5'-GTTTCCCCTAAACCGCTAGG-3' | 5'-AGCGAGAGTGGCAGAGGA-3' |
| Zeb1 (SYBR) | 5'-TGACAGAAAGGAAGGGCAAGA-3' | 5'-CAGGTGAGTAATTGTGAAAATGCAT-3' |
| Zeb2 (SYBR) | 5'-AGGCATATGGTGACGCACAA-3' | 5'-CTTGAACTTGCGGTTACCTGC-3' |
| Twist (SYBR) | 5'-GCCGGAGACCTAGATGTCATTG-3' | 5'-CACGCCCTGTTTCTTTGAATTT-3' |
| Snail (SYBR) | 5'-CCCAGTGCCTCGACCACTAT-3' | 5'-GCTGGAAGGTAAACTCTGGATTAGA-3' |
| TBP (SYBR) | 5'-ACGCCAGCTTCGGAGAGTT-3' | 5'-CCTCATGATTACCGCAGCAAA-3' |
| PTK7 (FAM) | Assay ID: Hs00897151_m1 (Thermo Fisher Scientific) | |
| TBP (FAM) | Assay ID: Hs00427620_m1 (Thermo Fisher Scientific) | |

**Table S2** Antibodies for immunocytochemistry and western blotting assays used in this study.

| **Antibodies for immunocytochemistry and western blot used in this study** | | | | |
| --- | --- | --- | --- | --- |
| Name | Catalogue | Company | Host | Application |
| rhodamine phalloidin | R415 | Invitrogen | rabbit | IF |
| phospho-paxillin (Y31) | ab32115 | Abcam | rabbit | IF, WB |
| phospho-FAK (Y397) | 44-625G | Invitrogen | rabbit | IF, WB |
| AlexaFluor555 | Z-25305 | Invitrogen | rabbit | IF |
| DAPI | D1306 | Invitrogen |  | IF |
| E-cadherin | 610181 | BD | mouse | WB |
| N-cadherin | 13116S | Cell Signaling | rabbit | WB |
| vimentin | 5741S | Cell Signaling | rabbit | WB |
| PTK7 | AF4499 | R&D | goat | WB |
| actin | MAB1501 | MerckMillipore | mouse | WB |
| α-tubulin | Gex102078 | Genetex | rabbit | WB |
